# A Patient-Centered Electronic Tool for Weight Loss Outcomes after Roux-en-Y Gastric Bypass

**DOI:** 10.1155/2014/364941

**Published:** 2014-03-20

**Authors:** G. Craig Wood, Peter Benotti, Glenn S. Gerhard, Elaina K. Miller, Yushan Zhang, Richard J. Zaccone, George A. Argyropoulos, Anthony T. Petrick, Christopher D. Still

**Affiliations:** ^1^Geisinger Obesity Research Institute, Geisinger Clinic, Danville, PA 17822, USA; ^2^Institute of Personalized Medicine, Penn State University College of Medicine, Hershey, PA 17033, USA; ^3^Department of Computer Science, Bucknell University, Lewisburg, PA 17837, USA; ^4^Weis Center for Research, Geisinger Clinic, Danville, PA 17822, USA; ^5^Department of Surgery, Geisinger Clinic, Danville, PA 17822, USA; ^6^Department of Gastroenterology, Geisinger Clinic, Danville, PA 17822, USA

## Abstract

*Background*. Current patient education and informed consent regarding weight loss expectations for bariatric surgery candidates are largely based on averages from large patient cohorts. The variation in weight loss outcomes illustrates the need for establishing more realistic weight loss goals for individual patients. This study was designed to develop a simple web-based tool which provides patient-specific weight loss expectations. *Methods*. Postoperative weight measurements after Roux-en-Y gastric bypass (RYGB) were collected and analyzed with patient characteristics known to influence weight loss outcomes. Quantile regression was used to create expected weight loss curves (25th, 50th, and 75th %tile) for the 24 months after RYGB. The resulting equations were validated and used to develop web-based tool for predicting weight loss outcomes. *Results*. Weight loss data from 2986 patients (2608 in the primary cohort and 378 in the validation cohort) were included. Preoperative body mass index (BMI) and age were found to have a high correlation with weight loss accomplishment (*P* < 0.0001 for each). An electronic tool was created that provides easy access to patient-specific, 24-month weight loss trajectories based on initial BMI and age. *Conclusions*. This validated, patient-centered electronic tool will assist patients and providers in patient teaching, informed consent, and postoperative weight loss management.

## 1. Introduction

Extreme obesity (BMI > 40 kg/m^2^) is increasing in prevalence, is rising faster than lower levels of obesity [1], and is associated with substantial morbidity and mortality. It is present in over 5% US adults and has been linked to numerous diseases including type 2 diabetes, hypertension, cardiovascular disease, and depression which reduce longevity and quality of life. The treatment options currently available include medical weight management (diet and exercise) and bariatric surgery. Currently, bariatric surgery is the most effective treatment for extreme obesity.

Despite an increasing number of patients who meet criteria for bariatric surgery, the number who actually undergoes bariatric surgery has leveled off in recent years (about 220,000 per year) [2]. This apparent stabilization of procedure numbers may be related to economic climate and reimbursement patterns. Other contributing factors include limited reimbursement for obesity management at the primary care level, variations in primary care physician comfort level with management of extreme obesity [3–5], and the media attention to the rare patients with adverse outcomes which may enhance reluctance among candidates for bariatric surgery [6].

Patients with extreme obesity struggle with the treatment decision between medical weight management and weight loss surgery [7, 8]. Patients spend an average of 3 years researching online for bariatric surgery before electing to have a surgical procedure performed [9]. In a recent study, less than half of the patients evaluated at a bariatric treatment center went on to have surgery in the same institution [7]. In addition, most patient education materials are generic and geared towards the “average” bariatric patient. Few informed consent teaching materials, if any, contain information that can be used to personalize patient-specific outcomes. Although bariatric surgery typically results in rapid weight loss, the amount of weight loss varies and often includes a modest amount of weight regain [10].

Many bariatric surgery candidates have unrealistic expectations regarding the weight loss that accompanies bariatric surgery. In one study, candidates for surgery expected to lose an average of 80% of excess weight and would be disappointed with an average weight loss of 52% of excess weight [11]. Similar findings of mismatched expectations were reported in another study of patients participating in a variety of weight loss treatments [12].

We sought to develop and validate a patient-centered weight loss guide for 24-month outcomes after Roux-en-Y gastric bypass (RYGB) using available clinical data previously associated with weight loss outcomes including preoperative BMI, [10, 13–17], age [10, 14, 18], and diabetes [10, 14, 16, 19]. We built an electronic tool designed to aid in the bariatric surgical decision making process, to establish realistic weight loss goals and to longitudinally monitor weight loss.

## 2. Methods

### 2.1. Study Population

Patients who entered the bariatric surgery program in the Center for Nutrition and Weight Management at Geisinger Clinic were offered participation in research program focused upon obesity. The study was approved by Institutional Review Board and all patients included in the study provided written informed consent. We selected patients who underwent RYGB surgery from January 2004 to February 2013 for the study. The preoperative bariatric surgery program typically lasted for 6 to 12 months and included a diet-induced weight loss target of 10% of body weight. Patients were scheduled for regular follow-up visits at the Geisinger Weight Management Clinic, typically occurring at 1 week, 2 weeks, 2 months, 5 months, 8 months, and 12 months after RYGB surgery and then every 6–12 months thereafter. The overall study population included the primary cohort and a validation cohort, which consisted of more recent, nonoverlapping cohort of patients who underwent RYGB at the same institution.

### 2.2. Study Variables

Data used for this study were obtained from several clinical sources and entered into a database of patients enrolled in the obesity research program. The data structure and acquisition methods were described elsewhere [20]. Briefly, the data were extracted from a comprehensive data warehouse, which contained a variety of data from the electronic health record (EHR, EpicCare her, Verona, WI). The data elements extracted from the research database for this study included the patient age, gender, preoperative diabetes status (yes/no), patient height and weight prior to surgery, and all postoperative weight measurements. Postoperative weight measures were carefully reviewed to identify and remove implausible or inconsistent values as described previously [20]. The baseline height was used to calculate BMI (kg/m^2^) for all weight measurements.

### 2.3. Statistical Analysis

Patient age, gender, preoperative BMI, and preoperative diabetes were evaluated for association with weight loss outcomes including 6-month excess weight loss and weight loss nadir [10]. Variables that had a consistent and moderate/large effect size were retained for further analysis. Quantile regression was used create longitudinal BMI curves after RYGB [21]. Separate regression models were used to estimate curves for each selected percentile of postoperative BMI (e.g., 25th, 50th, and 75th percentiles). The modeled regression equations were chosen to enable a flexible, nonlinear curve that may have differing estimates based on patient characteristics (in this example, age and baseline BMI):

(1) BMI=β0+β1∗BMI_baseline+β2∗AGE+β3∗TIME+β4∗TIME2+B5∗TIME3+β6∗BMI_baseline∗TIME+β7∗AGE∗TIME,

where BMI is body mass index, *β*_*x*_ is the parameter estimates from the quantile regression model, BMI_baseline is the patient's preoperative BMI (i.e., at time of initial preoperative visit, 6–12 months prior to surgery), AGE is years of age at initial preoperative visit, TIME is the number of months after surgery that the BMI was measured, TIME2 is the squared TIME, and TIME3 is cubic TIME. The resulting equations were used to estimate the BMI percentiles for selected combinations of BMI, age, and months after surgery. To assess the validity of the model results, data from an independent cohort of patients who underwent RYGB at the same institution were used. For this validation cohort, the quantile regression models were repeated and the resulting equations were compared against the primary cohort. SAS version 9.2 was used for statistical analysis.

## 3. Results

**Figure 1:**
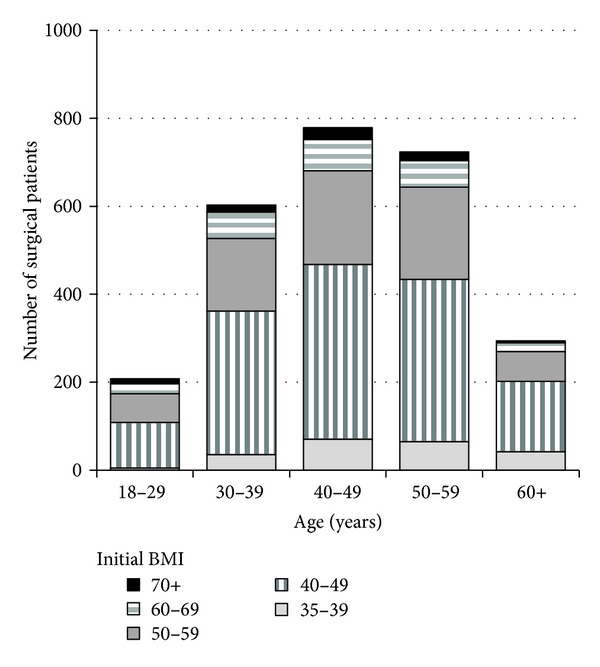
Distribution of initial BMI within various age intervals for patients that had RYGB (*n* = 2608).

**Figure 2:**
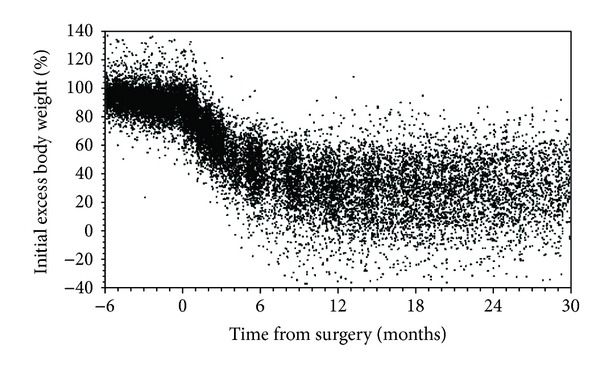
Scatter plot of the percent of excess weight lost for all patients from 6 months before surgery to 30 months after surgery.

We analyzed a cohort of 2,608 patients who had undergone RYGB surgery and who had an initial BMI >35 kg/m^2^. The mean age was 45.8 years (range 18–74), 81% were female, 97% were Caucasian, and the mean baseline BMI was 49.5 kg/m^2^ (range 35.0–94.3) (Table 1 and Figure 1). The median number of weight measurements during the first 36 months after surgery was 15 measures/patient and ranged from 1 to 149. For the 2,608 patients, there were 47,908 total weight measures during the 36 months after surgery (Figure 2). The percentage with at least 1 weight measure occurring >6 months, >12 months, and >24 months after surgery was 88%, 77%, and 53%, respectively. Patients with longer follow-up had similar characteristics to those of the overall population (Table 1).

In initial weight loss outcome analysis, preoperative BMI and age were significantly associated with 6-month weight loss (*P* value <0.0001 for both) and weight loss nadir. Both demonstrated moderate to large effect sizes and were selected for inclusion in the quantile regression models. Although diabetes was significantly associated with 6-month weight loss (*P* value = 0.0093) and weight loss nadir (*P* value <0.0001), the size of the effect was smaller than preoperative BMI and age and was not considered for further analysis. After accounting for age and BMI, patient gender was not associated with weight loss nadir (*P* value = 0.381) and was not considered for further analysis.

**Figure 3:**
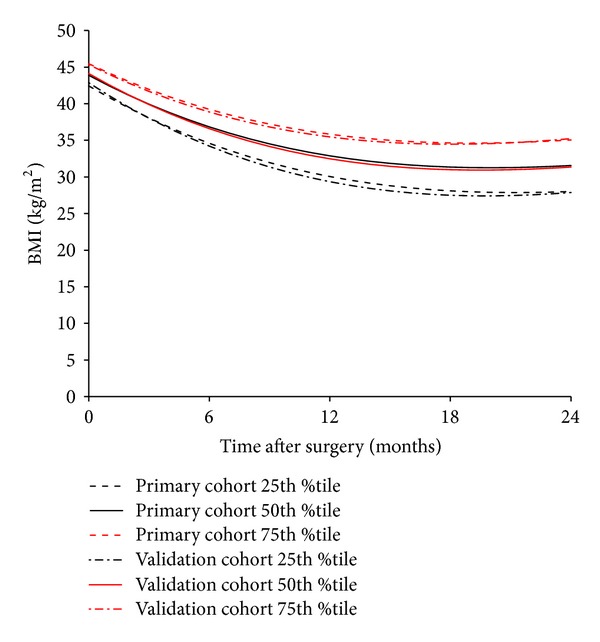
Comparison of estimated post-RYGB BMI between the primary cohort and the validation cohort using an age of 50 and an initial BMI of 50 kg/m^2^.

Quantile regression models were computed and resulted in formulas for calculating BMI over the 36 months after surgery (Table 2). These formulas were used to create lookup tables (Tables 3(a)–(c)) for the 25th, 50th, and 75th percentile of BMI at 6, 12, and 24 months after surgery stratified by initial BMI (from 40 to 70 kg/m^2^ in 5 kg/m^2^ intervals) and age (30, 50, and 70 years). A similar analysis was completed using the 378 patients in the validation cohort (Table 1), which resulted in similar quantile regression results (Figure 3).

**Figure 4:**
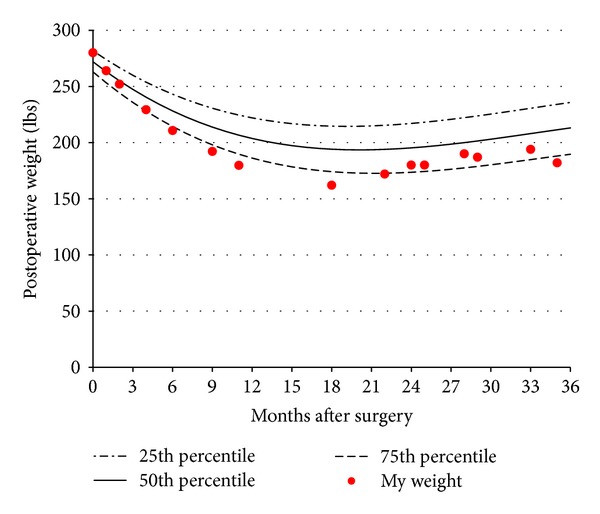
Example of a patient tracking their weight after surgery using the Get~2~Goal electronic tool.

In order to provide a useful and simple interface to the formulas and lookup tables, an electronic tool was developed. This tool allows the user to enter personalized patient characteristics including age, height, preoperative weight, and date of surgery (if completed but not required) which triggers the calculation for expected weight loss outcomes over the three years after surgery and displays them in a figure and a table. This electronic application (commonly referred to as “app”) allows the users to enter their postoperative weight values, which are overlaid on the expected weight loss outcomes figure (Figure 4). This provides a graphical presentation that allows the users to quickly determine if their weight is tracking with their preoperative expectations. Instructions for obtaining a free download of the app can be found on the Geisinger Health System webpage (http://www.geisinger.org) by searching for Get~2~Goal, The Geisinger Obesity Achievement Log.

## 4. Discussion

Weight loss outcomes after RYGB can vary substantially, making it difficult for patients and their care providers to characterize successful weight loss. In this study, we used readily available patient characteristics including preoperative BMI and patient age to develop an electronic tool to guide weight loss outcomes within the 2 years following RYGB. These results were validated using a nonoverlapping cohort of RYGB patients. Furthermore, the electronic tool allows for a simple and convenient user interface without having to access or understand the complex, underlying statistical formulas. This tool's simplicity assists in developing a patient-centered approach to facilitate decision making during patient preparation for bariatric surgery.

We have reported associations between weight loss outcomes, preoperative BMI [10, 13–17], and patient age [10, 14, 18]. These were included in the tool because these associations were replicated in this study and because the degree of the association was relatively large. Several diabetes metrics (e.g., HbA1c and medication use) have been shown to be associated with weight loss outcomes [10, 14, 16, 19]. We considered diabetes diagnosis for inclusion in the tool, which was significantly associated with weight loss outcomes but was not selected for inclusion in the tool for two primary reasons. First, the association with weight loss was of much lower magnitude than preoperative BMI and age. Second, the diagnostic criteria for diabetes vary among providers and the severity of diabetes will vary substantially among patients.

The unrealistic weight loss expectations of candidates for bariatric surgery as well as the 10–25% of surgical patients who struggle with weight maintenance and weight regain provide opportunity for improvement in patient education and selection for surgery. In the current environment of limited patient access to bariatric surgery, improved ability to teach and identify those patients who are prepared to be accountable and partners in the weight loss process should improve patient selection for surgery and may result in fewer weight loss failures. In addition, the use of such an electronic tool is likely to impact communication between patients and providers. For example, use during the preoperative decision making process could result in increased motivation and greater engagement with the clinical program. Similarly, this patient-centered weight loss tool can be used during postoperative clinic visits to monitor the weight loss trajectory and provide early documentation of behavioral and other factors which may be impacting weight loss outcomes (Figure 4). Future studies will need to be performed to determine the effects of the tool on patient motivation and engagement.

The large sample size, the depth of weight loss data, and length of postoperative follow-up [20] are strengths of this study, although only 53% of the population had 24-month weight data. However, the majority of those without 24-month data were patients who had undergone RYGB surgery relatively recently, that is, have not yet reached 24-months postoperative time point. Weaknesses include limitation of the results to a single institution and a single surgery type. Replication of the model with an external dataset and other surgery types is needed to validate the results and further studies to determine the effects of the tool on outcomes will be needed. In addition, the restricted racial and ethnic composition of the cohort may also limit its broader use in more diverse populations.

We are not aware of any other similar patient-centered tool targeted to bariatric surgical candidates and RYGB patients. Such tools may become important components of patient education and informed consent. The recent establishment of large clinical registries for bariatric surgery has facilitated the development of patient-centered risk scores [22, 23] and risk calculators [24, 25] which can be used with this tool for informed consent and in patient selection decision making. As additional risk metrics are developed (e.g., severity of comorbid conditions, mental health disorders, and behavioral conditions), more precise predictors of surgical and weight loss outcomes will be developed and may be incorporated into this tool. Future studies will need to be conducted to determine whether the use of patient-specific information for decision making, allowing for a more precise risk/benefit analysis, will enhance care delivery and long term outcomes.

## 5. Conclusion

We used a large cohort with deep clinical data to develop and validate an electronic patient-centered tool designed to assist patients and their providers in characterizing more realistic expectations for surgical weight loss and for postoperative weight loss management. This electronic nature of the technology will allow for efficient future modification to include other postoperative outcomes.

## Figures and Tables

**Table 1:** Demographic characteristics of primary study cohort by length of follow-up and of the validation cohort.

		Primary cohort				Validation cohort *N* = 378
		Total *N* = 2608	>6-month follow-up *N* = 2307	>12-month follow-up *N* = 2013	>24-month follow-up *N* = 1381	
Age	Mean (SD)	45.8 (11.2)	46.1 (11.1)	46.4 (11.1)	46.8 (10.9)	46.3 (11.0)
	Range	[18, 74]	[18, 74]	[18, 74]	[18, 72]	[19, 72]

Gender	Female, % (*N*)	81% (*n* = 2102)	81% (*n* = 1866)	81% (*n* = 1638)	82% (*n* = 1354)	78% (*n* = 293)
	Male, % (*N*)	19% (*n* = 506)	19% (*n* = 441)	19% (*n* = 375)	18% (*n* = 242)	22% (*n* = 85)

Race	Caucasian, % (*N*)	97% (*n* = 2534)	95% (*n* = 2243)	97% (*n* = 1961)	98% (*n* = 1354)	97% (*n* = 367)
	Black, % (*N*)	2% (*n* = 59)	2% (*n* = 50)	2% (*n* = 41)	2% (*n* = 22)	2% (*n* = 6)
	Other, % (*N*)	1% (*n* = 15)	1% (*n* = 14)	1% (*n* = 11)	<1% (*n* = 5)	1% (*n* = 5)

BMI	Mean (SD)	49.5 (8.7)	49.5 (8.8)	49.6 (8.7)	49.4 (8.6)	49.7 (8.2)
	Range	[35.0, 94.3]	[35.0, 94.3]	[35.0, 94.3]	[35.0, 94.3]	[35.5, 77.8]

**Table 2:** Results of quantile regression: formulas for selected percentiles of weight loss.

Percentile	Formula
25th (most weight loss)	25th %tile = 34.43 + 0.6878 ∗ (BMI—50) + 0.03358 ∗ (AGE—50) − 1.020 ∗ (TIME—6) + 0.04580 ∗ (TIME—6) ∗ (TIME—6) − 0.00055 ∗ (TIME—6) ∗ (TIME—6) ∗ (TIME—6) − 0.00725 ∗ (BMI—50) ∗ (TIME—6) + 0.001608 ∗ (AGE—50) ∗ (TIME—6)

50th	50th %tile = 36.71 + 0.7308 ∗ (BMI—50) + 0.02551 ∗ (AGE—50) − 0.906 ∗ (TIME—6) + 0.04298 ∗ (TIME—6) ∗ (TIME—6) − 0.00052 ∗ (TIME—6) ∗ (TIME—6) ∗ (TIME—6) − 0.00527 ∗ (BMI—50) ∗ (TIME—6) + 0.001542 ∗ (AGE—50) ∗ (TIME—6)

75th (least weight loss)	75th %tile = 39.11 + 0.7839 ∗ (BMI—50) + 0.02443 ∗ (AGE—50) − 0.790 ∗ (TIME—6) + 0.03937 ∗ (TIME—6) ∗ (TIME—6) − 0.00048 ∗ (TIME—6) ∗ (TIME—6) ∗ (TIME—6) − 0.00399 ∗ (BMI—50) ∗ (TIME—6) + 0.000927 ∗ (AGE—50) ∗ (TIME—6)

BMI: preoperative BMI; AGE: years of age at initial preoperative visit; TIME: months after surgery.

**Table:** (a)

Initial BMI	Age	Postoperative BMI percentile		
		25th	50th	75th
40	30	27.0	29.0	30.9
	50	27.7	29.5	31.4
	70	28.4	30.0	31.9

45	30	30.5	32.7	34.8
	50	31.2	33.2	35.3
	70	31.8	33.7	35.8

50	30	33.9	36.3	38.7
	50	34.6	36.8	39.2
	70	35.3	37.3	39.7

55	30	37.4	40.0	42.7
	50	38.0	40.5	43.2
	70	38.7	41.0	43.6

60	30	40.8	43.6	46.6
	50	41.5	44.1	47.1
	70	42.1	44.7	47.6

65	30	44.2	47.3	50.5
	50	44.9	47.8	51.0
	70	45.6	48.3	51.5

70	30	47.7	50.9	54.4
	50	48.4	51.5	54.9
	70	49.0	52.0	55.4

**Table:** (b)

Initial BMI	Age	Postoperative BMI percentile		
		25th	50th	75th
40	30	22.8	25.2	27.6
	50	23.6	25.9	28.2
	70	24.5	26.6	28.8

45	30	26.0	28.7	31.4
	50	26.8	29.4	32.0
	70	27.7	30.1	32.6

50	30	29.2	32.2	35.2
	50	30.1	32.9	35.8
	70	30.9	33.6	36.4

55	30	32.4	35.7	39.0
	50	33.3	36.4	39.6
	70	34.1	37.1	40.2

60	30	35.6	39.2	42.8
	50	36.5	39.9	43.4
	70	37.4	40.6	44.0

65	30	38.9	42.7	46.6
	50	39.7	43.4	47.2
	70	40.6	44.1	47.8

70	30	42.1	46.2	50.4
	50	42.9	46.9	51.0
	70	43.8	47.6	51.6

**Table:** (c)

Initial BMI	Age	Postoperative BMI percentile		
		25th	50th	75th
40	30	21.2	24.1	27.1
	50	22.4	25.2	27.9
	70	23.7	26.2	28.7

45	30	24.0	27.3	30.7
	50	25.2	28.3	31.5
	70	26.5	29.4	32.3

50	30	26.8	30.5	34.2
	50	28.0	31.5	35.0
	70	29.3	32.6	35.9

55	30	29.6	33.6	37.8
	50	30.8	34.7	38.6
	70	32.1	35.8	39.4

60	30	32.3	36.8	41.3
	50	33.6	37.9	42.2
	70	34.8	38.9	43.0

65	30	35.1	40.0	44.9
	50	36.4	41.1	45.7
	70	37.6	42.1	46.5

70	30	37.9	43.2	48.5
	50	39.2	44.2	49.3
	70	40.4	45.3	50.1
